# Integrated analysis of genome-wide DNA methylation and gene expression profiles identifies potential novel biomarkers of rectal cancer

**DOI:** 10.18632/oncotarget.11534

**Published:** 2016-08-23

**Authors:** Jiufeng Wei, Guodong Li, Jinning Zhang, Yuhui Zhou, Shuwei Dang, Hongsheng Chen, Qiong Wu, Ming Liu

**Affiliations:** ^1^ Department of General Surgery, The Fourth Affiliated Hospital of Harbin Medical University, Harbin, 150001, P.R. China; ^2^ Bio-Bank of Department of General Surgery, The Fourth Affiliated Hospital of Harbin Medical University, Harbin, 150001, P.R. China; ^3^ School of Life Science and Technology, State Key Laboratory of Urban Water Resource and Environment, Harbin Institute of Technology, Harbin, 150001, P.R. China

**Keywords:** DNA methylation, epigenetics, gene expression array, rectal carcinoma, molecular marker

## Abstract

DNA methylation was regarded as the promising biomarker for rectal cancer diagnosis. However, the optimal methylation biomarkers with ideal diagnostic performance for rectal cancer are still limited. To identify new molecular markers for rectal cancer, we mapped DNA methylation and transcriptomic profiles in the six rectal cancer and paired normal samples. Further analysis revealed the hypermethylated probes in cancer prone to be located in gene promoter. Meanwhile, transcriptome analysis presented 773 low-expressed and 1,161 over-expressed genes in rectal cancer. Correction analysis identified a panel of 36 genes with an inverse correlation between methylation and gene expression levels, including 10 known colorectal cancer related genes. From the other 26 novel marker genes, *GFRA1* and *GSTM2* were selected for further analysis on the basis of their biological functions. Further experiment analysis confirmed their methylation and expression status in a larger number (44) of rectal cancer samples, and ROC curves showed higher AUC than *SEPT9*, which has been used as a biomarker in rectal cancer. Our data suggests that aberrant DNA methylation of contiguous CpG sites in methylation array may be potential diagnostic markers of rectal cancer.

## INTRODUCTION

Colorectal carcinoma(CRC) is the third most common malignancy throughout the world [1]. Global statistics showed that in 2012 alone, over 1.36 million people were diagnosed with colorectal carcinoma, and approximately 693,900 people died from this disease [2]. Rectal cancers are reported to represent approximately 33% of CRC diagnoses [1]. Currently, the diagnosis of rectal cancer is primarily determined based on the clinical data and pathological analysis of patients [3]. However, successful early detection of rectal cancer patients is still hampered by the lack of highly sensitive and specific biomarkers. Therefore, the identification of biomarkers, including molecular biomarkers, for patient screening and early detection of CRC rectal cancer is a high priority.

Epigenetic abnormalities, including aberrant DNA methylation changes, have been reported to play an important role in various types of carcinogenesis, including rectal cancer. Given their important functions in cancer initiation and progression, methylation changes have been used as potential biomarkers for the early detection of cancers, including cervical, bladder, gastrointestinal, and lung cancer [4–7]. Several aberrant methylated genes such as *SEPT9* and *SFRP2* have been reported as biomarkers [8, 9]. However, the sensitivity and specificity of these molecular methylation biomarkers are still not satisfied. Therefore, despite the long list of aberrantly methylated genes in rectal cancer patients, promising DNA methylation biomarkers have not yet reached to the clinical utility.

Recent progress in high-throughput DNA technologies, including DNA microarrays, has increased the capability of interrogating genome-wide DNA methylation status in human cancer [10]. For example, the Illumina 450K microarray is one of the most powerful tools available for displaying differential DNA methylation, and represents a significant improvement in the detection of CpG site density, as it includes analysis of both CpG island (CGI) sites and non-CGI sites [11]. A number of DNA methylome studies have been reported in a variety of primary cancers, including rectal cancer. However, few studies have been managed to vigorously validate the methylation alterations of the candidate genes at the molecule level in numerous cancer samples [12, 13].

The purpose of this study was to identify new molecular diagnostic biomarkers for rectal cancer by mapping DNA methylation and transcription profiles in tumor tissue from six confirmed cases and paired normal tissue samples. The Illumina 450K microarray was chosen to map genome-wide DNA methylation profiles, with comparative analysis to identify a large number of differentially methylated CpG sites in rectal cancer and normal rectal tissue. A further aim of the study was to evaluate the molecular findings in 44 available paired rectal tumor and normal tissue samples, to identify whether novel potential biomarker genes for rectal cancer could be identified by their expression and methylation status. We demonstrated that the methylation status of *GFRA1* and *GSTM2* could be used as potential biomarkers for the screening of rectal cancer.

## RESULTS

### Whole genome analysis of differential DNA methylation

To identify differentially methylated probes related to rectal cancer, whole genome DNA methylation analysis was performed in six pairs of rectal cancer and normal tissues with the Illumina 450K beadchip array. Using this method, 18,568 probes were identified with significant methylation differences between the six rectal cancer and six normal samples (paired Wilcoxon's test, *P*<0.05).

**Figure 1:**
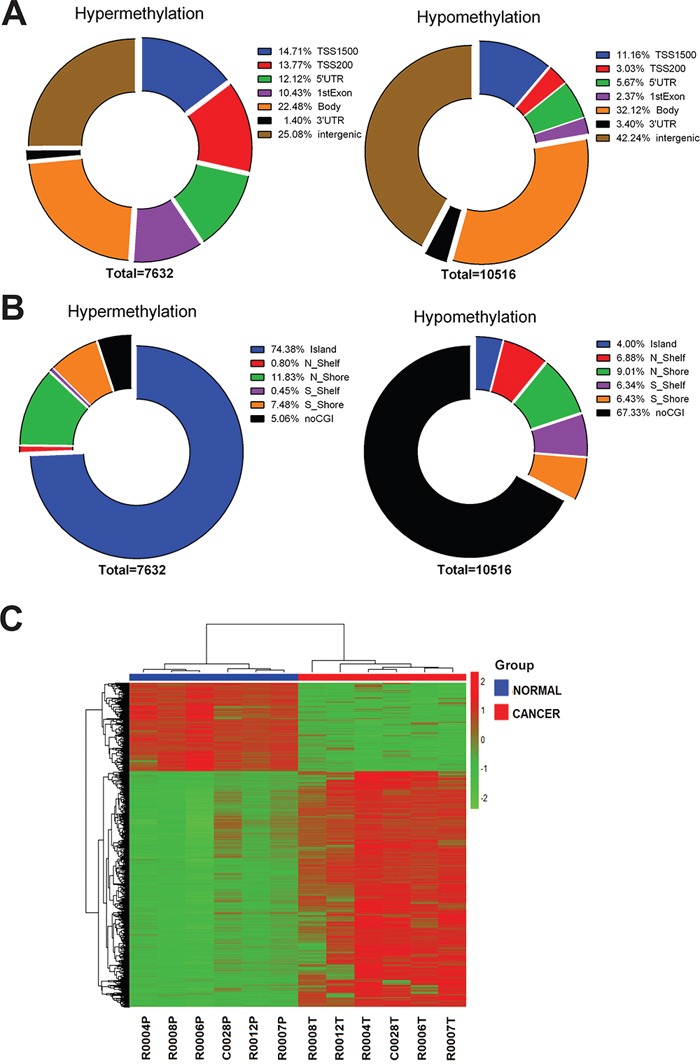
Distribution of probes with significant rectal cancer-related differential methylation changes in the human rectal carcinoma genome **A.** Pie chart shows the distribution of the hypermethylated and hypomethylated CpG sites over TSS200, TSS1500, 5′UTR, 1st exon, 3′UTR and intergenic. The percentage of CpG counts is indicated in the diagrams. **B.** Pie chart shows the distribution of the hypermethylated and hypomethylated CpG sites over CpG islands, CpG shores, CpG shelves and non CGI regions. The percentage of CpG counts is indicated in the diagrams. **C.** Hierarchical clustering was performed using significantly differentially methylated probes and a heatmap was made. Red indicates high methylation; green low. Above the columns cancers are marked with red and normal samples with blue.

Of the differentially methylated probes, 7632 showed hypermethylation (43.4%, [rectal cancer]>[normal tissue]), while 10516 were hypomethylated (56.6%, [rectal cancer]<[normal tissue]) (Figure 1A). More than one-fifth of the hypomethylated probes (22.2%) were found within the promoter region (TSS 1500, TSS 200, 5′-UTR and 1st exon). More than half of the hypermethylated probes (51%) were within the promoter region (Figure 1A). Two-thirds (67.3%) of the hypomethylated probes were found to be non CpG island (CGI) sites. Most the hypermethylated probes (94.9%) were within CpG island or around CpG island (shelf, shore), indicating that hypomethylation occurred mainly in non CGI regions (Figure 1B). The differentially methylated probes between cancer tissues and normal tissues are represented by the heatmap shown in Figure 1C.

### Aberrantly expressed genes induced by DNA methylation in rectal cancer

**Figure 2:**
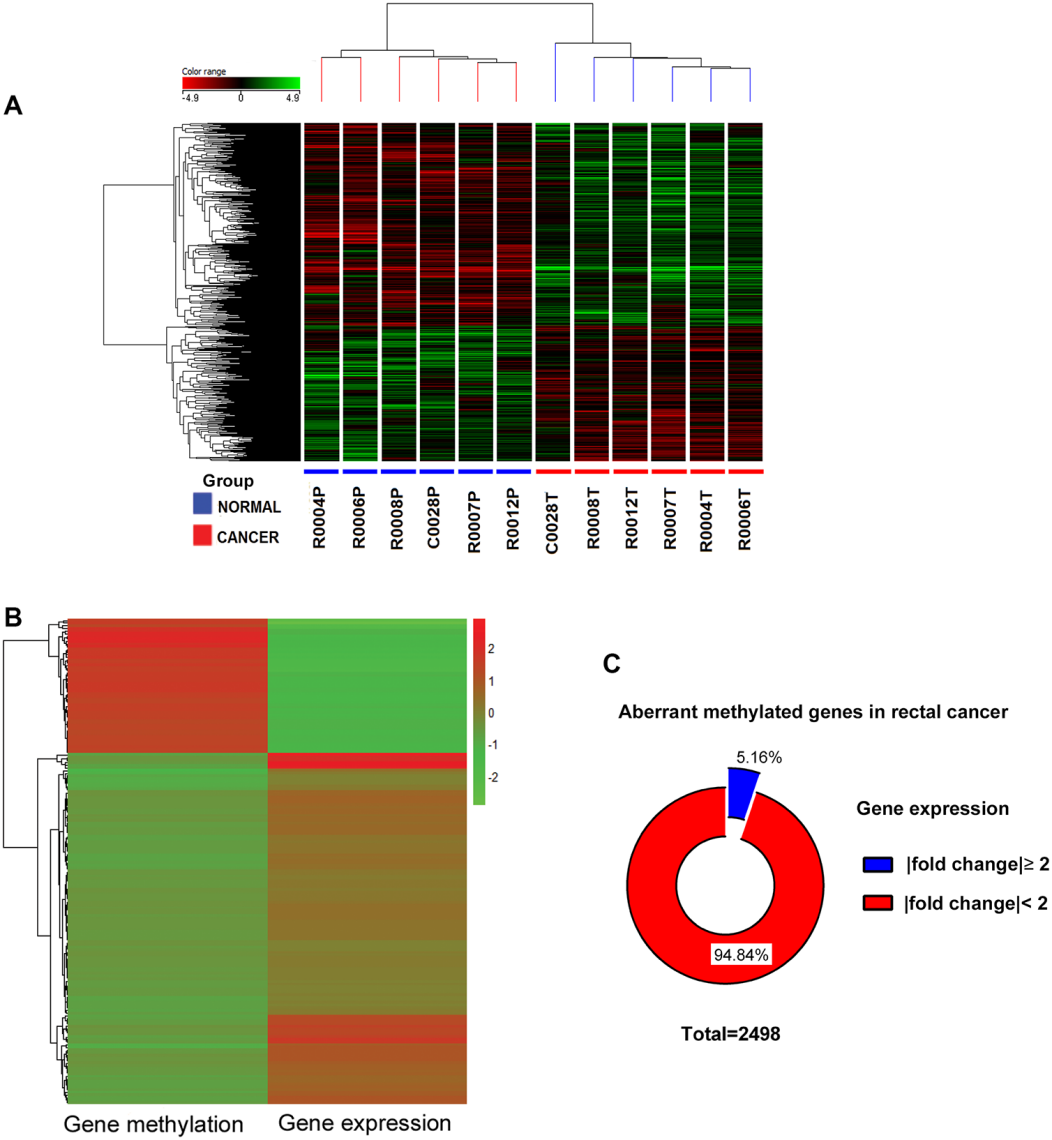
Integrated analysis of genome-wide DNA methylation and gene expression profiles **A.** Supervised hierarchical cluster analysis showing differentially expressed genes in rectal cancer. Genes in red indicate overexpression; those in green indicate underexpression. Under the columns cancers are marked with red and normal samples with blue. **B.** A heatmap was made by genes with inverse relation between methylation and expression, Red indicates high level; green low. **C.** Pie chart showing the gene expression changes of 2498 aberrant methylated genes in rectal cancer compared with adjacent normal tissues.

To identify potential molecular biomarker candidates, Illumina HT12v4 gene expression array was conducted with the same six pairs of rectal cancer and normal tissues. Transcriptome profiling resulted in 773 under-expressed and 1,161 over-expressed genes (Figure 2A).

Integrated analysis of genome-wide DNA methylation and gene expression profiles were performed. We focused specifically on gene promoters, which are prime candidates for epigenetic regulation. Since transcriptional alteration does not require aberrant methylation of the entire CpG island or the entire promoter, the average of the β-values of the only differential methylated CpG sites in the TSS 200, TSS 1500, 5′-UTR and 1st exon regions were used as a proxy for the gene methylation level. Genes with an inverse correlation between methylation and expression were selected for further investigation (Figure 2B). Only 5.16% aberrant methylated genes correlated with inverse expression (Figure 2C). Thirty-six genes with the number of differentially methylated CpG site in the promoter more than two are listed in Table 1.

**Table 1:** List of 36 genes with the number of differentially methylated CpG site in the promoter more than 2

gene	probe number*	Mean β value		Δβ	gene	probe number*	Mean β value		Δβ
		normal	cancer				normal	cancer	
**EYA4**	**27**	**0.151**	**0.469**	**0.318**	**FRZB**	**4**	**0.236**	**0.487**	**0.251**
**GFRA1**	**20**	**0.134**	**0.472**	**0.338**	**GALR1**	**4**	**0.255**	**0.496**	**0.241**
**FOXI2**	**15**	**0.291**	**0.579**	**0.288**	**PMEPA1**	**4**	**0.754**	**0.457**	**-0.297**
**SLITRK1**	**9**	**0.336**	**0.610**	**0.274**	**RARRES2**	**4**	**0.396**	**0.639**	**0.243**
**STOX2**	**9**	**0.118**	**0.378**	**0.260**	**SLC6A5**	**4**	**0.376**	**0.605**	**0.229**
**CNRIP1**	**8**	**0.182**	**0.531**	**0.349**	**AZGP1**	**3**	**0.718**	**0.453**	**-0.265**
**SFRP1**	**8**	**0.292**	**0.549**	**0.257**	**C10orf81**	**3**	**0.641**	**0.417**	**-0.224**
**ADHFE1**	**7**	**0.133**	**0.627**	**0.494**	**FAM110A**	**3**	**0.787**	**0.542**	**-0.245**
**C2orf40**	**6**	**0.265**	**0.528**	**0.263**	**GLRA3**	**3**	**0.194**	**0.445**	**0.251**
**KCNC2**	**6**	**0.279**	**0.572**	**0.293**	**GSTM2**	**3**	**0.216**	**0.514**	**0.298**
**KCNQ1**	**6**	**0.670**	**0.481**	**-0.189**	**HSD11B1**	**3**	**0.700**	**0.446**	**-0.254**
**LONRF2**	**6**	**0.110**	**0.498**	**0.388**	**MAL**	**3**	**0.173**	**0.471**	**0.298**
**MEST**	**6**	**0.762**	**0.505**	**-0.257**	**PHACTR3**	**3**	**0.715**	**0.454**	**-0.261**
**RALYL**	**6**	**0.287**	**0.550**	**0.263**	**SST**	**3**	**0.293**	**0.533**	**0.240**
**HKDC1**	**5**	**0.782**	**0.533**	**-0.249**	**TMEFF2**	**3**	**0.157**	**0.475**	**0.318**
**KCNIP4**	**5**	**0.190**	**0.443**	**0.253**	**TNFRSF8**	**3**	**0.815**	**0.581**	**-0.234**
**SORCS1**	**5**	**0.223**	**0.524**	**0.301**	**TUSC3**	**3**	**0.244**	**0.464**	**0.220**
**CBLN2**	**4**	**0.236**	**0.495**	**0.259**	**ZNF655**	**3**	**0.056**	**0.279**	**0.223**

* the number of differentially methylated probes in gene promoter region

Ten of the 36 (*EYA4* [14], *FOXI2* [15], *CNRIP1* [16], *SFRP1* [17], *ADHFE1* [18], *C2orf40* [19], *MAL* [20], *PHACTR3* [21], *SST* [20], *TMEFF2* [15]) were known as rectal cancer related genes with aberrant methylation. Eleven other genes identified (*GFRA1* [22], *SLITRK1* [23], *KCNQ1* [24], *MEST* [25], *FRZB* [26], *GALR1* [7], *PMEPA1* [27], *RARRES2* [28], *GSTM2* [29], *TNFRSF8* [30], *TUSC3* [31]) have been previously reported as genes with aberrant methylation in other forms of cancer. The CpG identity and average methylation level of the 36 genes identified are shown in Supplemental Table 1.

### Validation of DNA methylation status and expression pattern of selected novel rectal cancer genes

**Figure 3:**
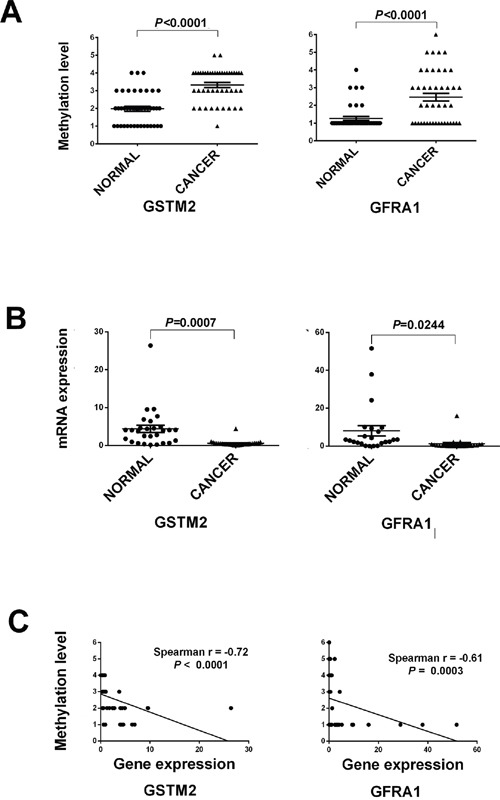
The strip plot shows the different methylation level and mRNA expression level in two groups and their integrated analysis of two genes **A.** The strip plot shows the significantly different methylation level of two genes, GFRA1 and GSTM2. Methylation level 1 to 6 represents the methylation intervals: 0–10%, 11–25%, 26–50%, 51–75%, 76–85%, and 86–100%. **B.** The strip plot shows the significantly mRNA expression fold change of two genes. **C.** Pearson correlations of two genes were used to measure linear relationships between gene methylation (y axis) and gene expression level (x axis).

To confirm differential methylation in the Beadchip data, *GFRA1* and *GSTM2* genes were selected for further analysis on the basis of their biological functions, the level of aberrant methylation, and their novel description in rectal cancer. MS-HRM of two hypermethylated genes (*GFRA1* and *GSTM2*) were conducted to validate an additional 44 pairs of rectal cancer and normal tissue samples (Table 2). Based on methylation-sensitive high-resolution melting curve analysis, methylation levels were scored in the intervals: 0–10%, 11–25%, 26–50%, 51–75%, 76–85%, and 86–100%. Differences in methylation could be validated in all two genes between two groups (*P*<0.05) (Figure 3A). Furthermore, the rectal cancer samples showed a significantly greater level of hypermethylated *GFRA1* and *GSTM2* when compared with the normal samples (*P*<0.05). The methylation states of these two promoters were further analyzed to determine if they were associated with any clinicopathological features of rectal cancer. MS-HRM results showed that the methylation states of the *GFRA1* and *GSTM2* promoters were not associated with rectal cancer stage (data not shown), but that aberrant methylation of these two genes may play roles in the pathogenesis of rectal cancer.

**Table 2:** Clinicopathological characteristics of rectal cancer patients

		Screening set (array)	Validation set (MS-HRM)
Gender	Female	4	21
	Male	2	23
Age	Mean	65.5	63.86
	Range	50-76	46-85
Differentiation	Well/moderate	5	39
	Poor	1	5
Stage	I	0	4
	II	3	21
	III	3	17
	IV	0	2
T	1	0	2
	2	0	4
	3	6	30
	4	0	8
N	0	3	26
	1	1	12
	2	2	6
M	0	6	42
	1	0	2

To determine whether methylation changes in these promoters affected gene expression, the mRNA levels of *GFRA1*, and *GSTM2* were quantified by quantitative RT-PCR on a matched tissue sample set consisting of 26 samples from the validation set. Significant differential mRNA levels between paired rectal cancer and normal tissue were observed (*P*<0.05, Figure 3B). The qRT-PCR results showed that mRNA expression levels of all two genes *GFRA1* and *GSTM2* were inversely correlated with the prevalence of methylation in their promoters. Pearson's correlation analysis was further performed to assess the relationship between gene methylation and gene expression levels. The expression levels of *GSTM2* and *GFRA1* were inverse correlated with methylation levels of the three genes. Pearson's correlation analysis was performed to assess the relationship between gene methylation and gene expression levels, with correlation values ranging from −0.72 to −0.61 (P < 0.0001 to 0.0003, Figure 3C).

Selected CpG sites and genes were then analyzed on a publicly-available tool, MEXPRESS (http://mexpress.be), with a methylation and expression data set of 394 colorectal carcinomas(CRC) from The Cancer Genome Atlas (TCGA, http://tcga.cancer.gov/) [32]. The methylation status of these samples was further determined using the 450K methylation array, with results showing consistency with our study data (Supplemental Figure 1).

### Evaluation of selected aberrant DNA methylation as potential diagnostic markers

**Figure 4:**
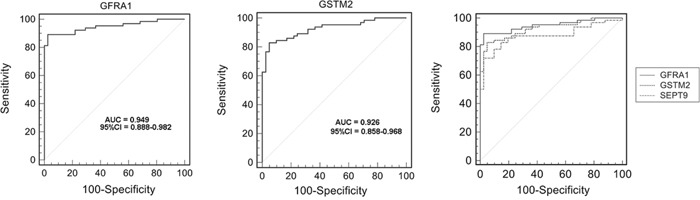
Receiver operating characteristic (ROC) curve showing a high-level of discrimination between normal tissue and colorectal carcinoma (CRC) tissue samples based on *GFRA1/GSTM2/SEPT9* methylation using dataset GSE48684

To evaluate selected aberrant DNA methylation of *GFRA1* and *GSTM2* as potential molecular biomarkers, ROC curve data were obtained by plotting the rate of sensitivity versus 100-specificity with dataset GSE48684 containing colon cancers and rectal cancers [33], which is available from the Gene Expression Omnibus (GEO) website. As shown in Figure 4 and Supplemental Table 2, these results showed that *GFRA1* and *GSTM2* methylation were able to discriminate between CRC tissue and normal control tissue with an area under the curve (AUC) of 0.949 and 0.926, respectively. At the cutoff values of 0.160 and 0.421, the sensitivities and specificities of *GFRA1* and *GSTM2* were 89.06% and 97.56%, 82.81% and 95.12%, respectively. The ROC curve of *SEPT9* methylation was analyzed with the same dataset. The selected genes, *GFRA1* and *GSTM2*, showed higher AUC than *SEPT9*.

## DISCUSSION

Since genome-wide changes of DNA methylation occurred in the beginning of carcinogenesis, DNA methylation has been considered as one of the most powerful promising biomarkers for early detection and screening in cancer [34, 35]. Despite the recent reports of large number of global DNA methylation studies for different tumor types, these studies have not performed follow-up studies to validate the candidate genes discovered from the genome-wide analyses [29, 36–38]. However, this study has presented a comprehensive and quantitative characterization of DNA methylation biomarkers in rectal cancer.

Previous studies on DNA methylation of rectal cancer have been performed using enrichment-based DNA sequencing approaches, which, although powerful analytical tools, can have low statistical power in CpG-poor genomic regions and relatively low resolution [39]. Aberrant methylation of CpG promoter sites may contribute to the regulation of gene expression, even for promoters with low CpG density, hypermethylation can suppress expression [40]. The 450K BeadChip array can assess more than 485,000 methylation sites per sample, covering 96% of CpG islands and 99% of Reference Sequence (RefSeq) genes, with an average of 17 CpG sites per gene region [11]. This methodology enabled this study to characterize differentially methylated regions involved in the pathogenesis of rectal cancer and to identify novel DNA methylation biomarkers that have not previously been associated with aberrant methylation in rectal cancer. This study showed rectal cancer specific methylation patterns consisting of 18,568 CpG sites that were significantly different from the paired normal tissues. Most of the hypermethylated CpG sites (74.38%) were located in the CpG island (CGI), while many of the hypomethylated CpG sites (67.33%) were located in the non-CGI region. These results are consistent with previously published studies [12, 41].

Clinically relevant aberrant methylation of a specific DNA locus may serve as a surrogate biomarker which is not is not always linked with changes in gene expression. However, the best-validated markers are expected to be those that show a good correlation between DNA methylation and gene expression. In this study, the DNA methylation data was integrated with gene expression profiles of the same rectal tumor to distinguish between DNA methylation events of potential functional significance (driver events) and events that did not contribute to tumorigenesis (passenger events), as methylation of a few gene-specific core CpG sites are most likely be sufficient for transcription [40]. Because the biological and clinical consequences of aberrant methylation of the promoter CpG island are strongly dependent on the expression status of the core regions, the average of the β-values of the differentially-methylated promoter CpG sites were set as a proxy for the gene methylation level. It may be more accurate, effective, and biologically relevant to use this approach for possible random aberrant methylation of a single CpG site. Finally, the cross-validated correlation between methylation level and expression of target genes indicates that the relevant differentially methylated loci were detected in rectal cancer in this study.

The findings of this study have confirmed some of the previous findings of candidate biomarker genes, but also provide some novel loci that show differential methylation in rectal cancer. Concerning the candidate genes identified as potential molecular biomarkers for rectal cancer, GFRA1, and GSTM2, aberrant methylation of GSTM2 has previously been identified in prostate cancer, breast cancer and oral squamous cell carcinoma [29, 42, 43]. GSTM2 belongs to the glutathione-S-transferases (GSTs) superfamily. GSTs catalyze the conjugation of the glutathione conjugation to a wide range of electrophilic substrates involved in detoxification processes [44]. GSTM1 is also a key member of the GSTs superfamily and has previously been associated with increased cancer risk in as a homozygous deletion polymorphism in some ethnic groups. GSTM2 can compensate for the loss of the GSTs enzyme due to the absence of GSTM1 under normal conditions [45]. It can be hypothesized that hypermethylated GSTM2 alone or with the absence of GSTM1 maybe promote carcinogenesis in rectal cancer, but this hypothesis requires confirmation with further studies.

GFRA1 is a cell surface GDNF/neurturin receptor and a tyrosine kinase that is usually expressed in the nervous system and kidney. GFRA1 protein is the key component of the GDNF-GFRA1-RET pathway, which can capture GDNF and deliver it to the RET receptor on the cell surface to activate the signal. This gene is over-expressed in gut neural crest stem cells and in many cancers [46, 47]. GFRA1 protein released by the microenvironment can promoted may enhance cancer cell PNI through activation of RET [48]. The aberrant methylation of GFRA1 has been reported in lung cancer and gastric cancer [22, 47]. The findings of the present study for hypermethylation of GFRA1 and GSTM2 may provide an explanation for their low-expression in rectal cancer. In addition this study showed that the methylation status of GFRA1 or GSTM2 was associated with rectal cancer.

In combination with the findings of the cross-validation results and the TCGA dataset for colorectal carcinoma (CRC) and the ROC curves which showed that *GFRA1* and *GSTM2* had a greater AUC than *SEPT9*, this study supports the possible role for these two genes as potential diagnostic molecular biomarkers for CRC. However, cross-validation studies remain to be done on the methylation status of these genes in circulating plasma DNA or fecal DNA in patients with CRC.

In conclusion, the findings of this study support the method of aberrant DNA methylation of contiguous CpG sites using methylation arrays to detect potential molecular tumor biomarkers and indicate that further studies should be done on the role of *GFRA1* and *GSTM2* as potential molecular biomarkers of rectal cancer.

## MATERIALS AND METHODS

### Subjects

Fifty pairs of rectal cancer tissues and adjacent normal tissues were obtained from the Bio-Bank of the Department of General Surgery, the Forth Affiliated Hospital of Harbin Medical University. Inclusion criteria for patients in the study were that no other cancers than rectal cancer were present at the time of surgical resection, there was no history of hereditary rectal cancer, and no radiotherapy or chemotherapy treatment had been given prior to surgical resection. The clinicopathological characteristics of each patient in the study are summarized in Table 2. This study has ethical approval and informed consent was obtained. The diagnosis of rectal cancer was confirmed for each patient from surgical histopathology reports. Fresh tissue samples were collected immediately following surgery, frozen in liquid nitrogen and stored at −80°C. The adjacent normal colorectal tissues were sampled at a distance of no less than 5cm from the tumor, and the presence of normal tissue was confirmed by histology. All rectal tissue samples used in this study were evaluated by a surgical pathologist to confirm the diagnosis of primary rectal cancer and to ensure that the tumor samples used in the study contained > 60% tumor tissue, preferably without necrosis.

### DNA extraction, bisulfite conversion and 450K microarray

Genomic DNA was extracted using standard phenol-chloroform techniques and quantified using spectrophotometry. Genomic DNA from all samples was treated with EZ DNA Methylation Kit (Zymo Labs, Irvine, CA, USA) according to the manufacturer's protocol. Briefly, 500 ng of bisulfite-converted DNA was hybridized onto the Infinium Human Methylation 450 BeadChip array following the Illumina Infinium HD Methylation protocol, with data processed with the Methylation Module of GenomeStudio v1.8 software.

The methylation levels of CpG sites were calculated as β-values. The β-value is a continuous variable of between 0 and 1. A β value of 0 corresponds to no methylation while a value of 1 corresponds to 100% methylation at the specific CpG site measured. Unreliable probes were first removed with a detection P-value>0.05. Also, CpG sites were removed on the X and Y chromosome, binding multiple genomic regions, containing single nucleotide polymorphisms (SNPs). The methylation data were deposited in the National Center for Biotechnology Information (NCBI) Gene Expression Omnibus (GEO) database repository of high throughput gene hybridization data (GEO: GSE75550).

### Differential methylation analysis

Comparison of the averaged methylation values was made between clinical groups at the CpG site level using Wilcoxon's test for paired samples. Benjamini-Hochberg method was used to calculate the false discovery rate (FDR). The following criteria were used: β-difference > 0.2 and a FDR-corrected *P* value < 0.05. These same criteria were used to calculate the methylation difference among the CpG site level variants identified. Since the promoter region and 1st exon both play critical roles in transcriptional regulation, the average of the β-values of differential CpG sites in the transcription start site (TSS) 200, TSS 1500, 5′- untranslated regions (UTR) and 1st exon was used as a proxy for the gene methylation level.

### RNA extraction and gene expression array

RNA was extracted from serial rectal tissue cryosections using RNAiso plus (Takara, Otsu, Japan) and quantified by spectrophotometry. Gene expression analysis was performed through the Illumina HumanHT-12 v4 Expression BeadChip. Reverse transcription of total RNA for gene expression analysis and cRNA synthesis with simultaneous biotin labeling were conducted with the Illumina TotalPrep-96 RNA Amplification Kit (Ambion, Darmstadt, Germany).

The cRNA was hybridized overnight to Human Gene Expression v12 array (Illumina, San Diego, CA) according to the manufacturer's instructions. Batch effects were avoided by labeling all samples and scanning them in random order. The data from Infinium HumanMethylation450 BeadChip Array were scanned using the Illumina BeadChip Array Reader. Infinium expression data were processed with Genome Studio Gene Expression Module v1.0 software. Linear modeling of the transformed data was performed using Limma in R. Only genes with a fold change ≥ 2 and a FDR corrected *P* value < 0.05 between any of the groups were considered as significantly differentially expressed.

### Quantitative real-time RT–PCR (qRT–PCR)

Reverse transcription reactions were performed using RT reagent kit (Takara, Otsu, Japan) according to the manufacturer's protocol. qRT–PCR was performed in triplicates using an Applied Biosystems (ABI) 7500 Real-Time PCR System (Applied Biosystems, Weiterstadt, Germany) using the relevant (SYBR Green) Master Mix (Takara Otsu, Japan). The GAPDH gene was used as a normalization gene for analysis of normal mucosa and rectal cancer specimen sets.

### Methylation sensitive high resolution melting curve (MS-HRM)

The molecular biomarker tissue validation set consisted of tissue samples from 44 patients with histologically-confirmed rectal cancer. Amplification of bisulfite modified DNA was performed in triplicates with primers designed according to guidelines published by Wojdacz *et al* [49]. The primers are listed in Supplementary Table 3.

PCR was performed with LightCyclerVR 480 High-Resolution Melting Master Mix (Roche, Hvidovre, Denmark) in a total volume of 10 μl. Standard curves from bisulfite modified templates were prepared by mixing 100% methylated DNA (CpGenomeTM Universal Methylated DNA, Qiagen) with a background of unmethylated DNA (Qiagen). The standard curve ranged from 0% methylated DNA, through 1, 5, 10, 25, 50, 75, 85 to 100% methylated bisulfite converted DNA. Standard curves and no template controls were included in each experimental run. The PCR reaction and high resolution melting curve analysis was performed essentially using a LightScanner instrument (Idaho Technology, Salt Lake City, Utah, USA). MS-HRM data were normalized with the LightScannerVR Instrument and Analysis Software to compensate for varying starting fluorescence levels. Patient data were classified by their different methylation categories by two independent observers, based on the standard curves (Supplemental Figure 2). The Kappa coefficient was calculated to evaluate the inter-observer agreement of the scored methylation levels for all two analyzed genes. The coefficients ranged from 0.91 to 1.0, indicating very good to excellent agreement.

### Statistical analysis

The statistical analysis was conducted using GraphPad Prism 6 software (La Jolla, CA, USA) and MedCalc version 10.1.6 (MedCalc Software, Mariakerke, Belgium). Paired Student's t-test was used to compare gene expression levels between rectal cancer and normal tissue samples. Associations between DNA methylation status and clinico-pathological features of the patients were analyzed by an unpaired t-test (Student's t-test or Welch's t-test) and Fisher's exact test. All reported *P*-values were two-sided, with *P* < 0.05 being considered statistically significant. Spearman's rank correlation coefficients were used to assess correlations between methylation and gene expression, *P* values < 0.05 were considered statistically significant. Receiver operating characteristic (ROC) analysis was performed by MedCalc statistical software.

## SUPPLEMENTARY FIGURES AND TABLES





## Abbreviations

CRC: colorectal carcinoma
CGI: CpG island
FDR: false discovery rate
GEO: Gene Expression Omnibus
GSTs: glutathione-S-transferases
MS-HRM: methylation sensitive high resolution melting curve
NCBI: National Center for Biotechnology Information
TSS: transcription start site
TCGA: The Cancer Genome Atlas
UTR: untranslated regions
ROC: receiver operating characteristic
