# Supplementary material for: Integrated analysis of genome-wide DNA methylation and gene expression profiles identifies potential novel biomarkers of rectal cancer
Source: Oncotarget. 2016 Aug 23;7(38):62547–58. doi: 10.18632/oncotarget.11534 (PMC5308745; doi:10.18632/oncotarget.11534)
Supplement: Supplementary file 1 [file oncotarget-07-62547-s001.pdf]

Integrated analysis of genome-wide DNA methylation and gene expression profiles identifies potential novel biomarkers of rectal cancer

SUPPLEMENTARY FIGURES AND TABLES

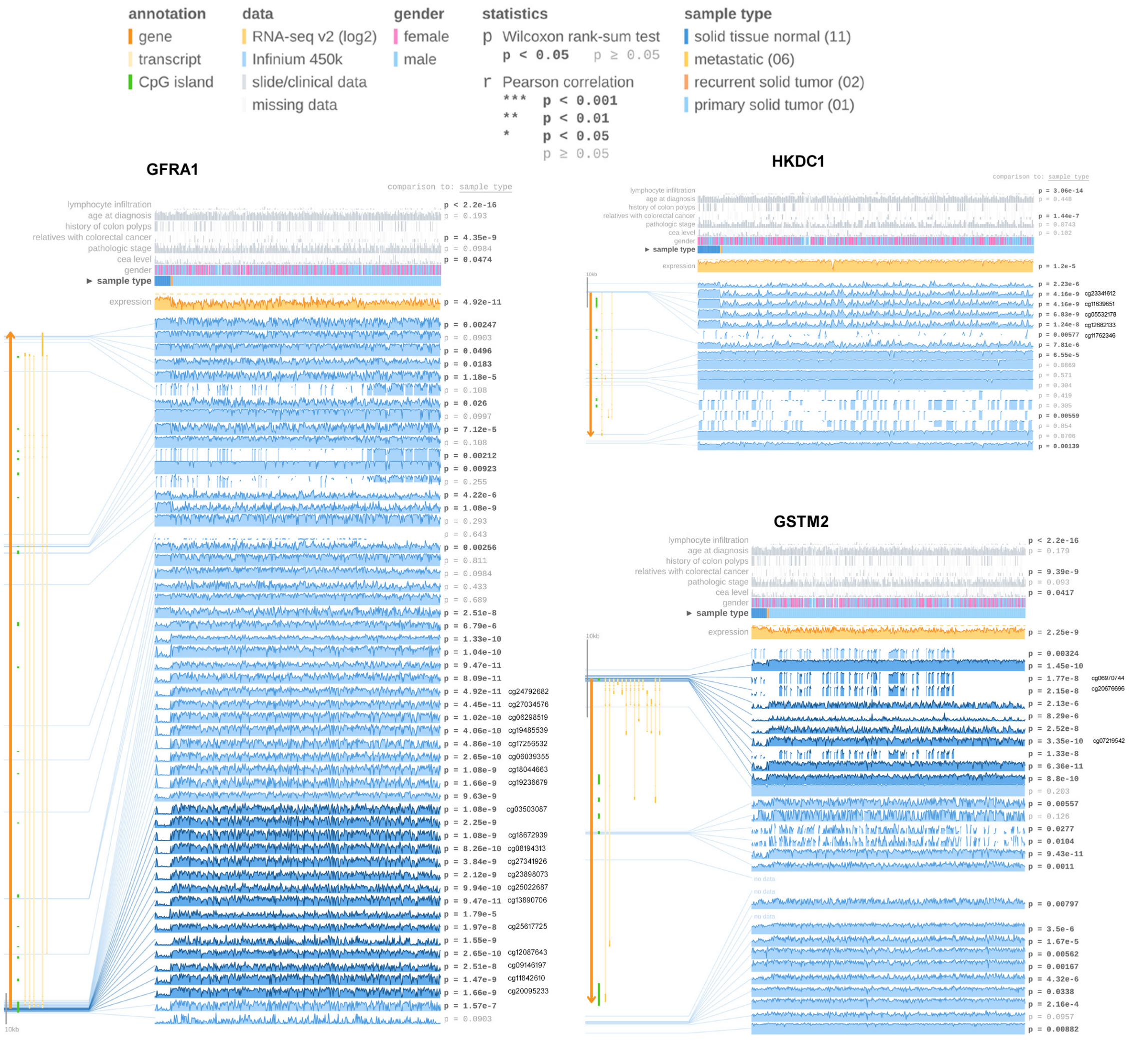

Supplementary Figure S1: Visualization of the TCGA data for three genes in colorectal adenocarcinoma using MEXPRESS. The samples are ordered by sample type, revealing clear differences in expression and methylation. The CpG ids with different methylation level screened in this study are marked behind.

A

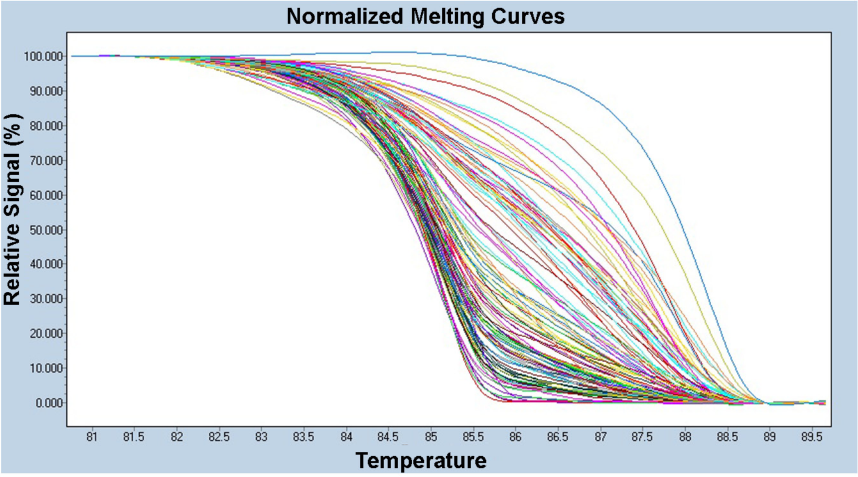

GSTM2

B

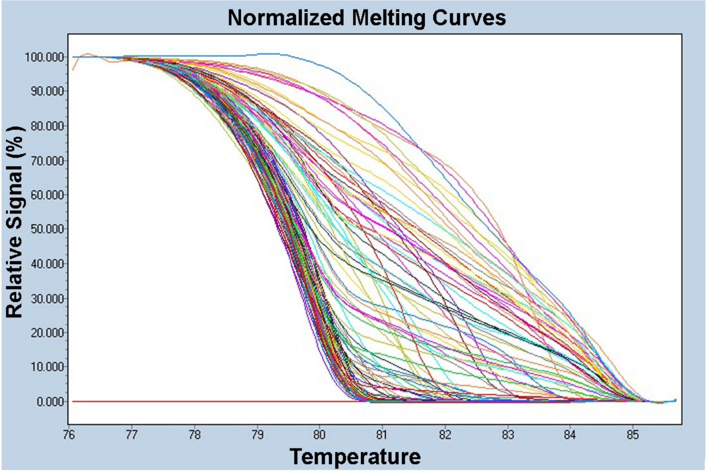

GFRA1

Supplementary Figure S2: Normalized MS-HRM standard curves and different plots of three aimed genes. Methylation levels of *GSTM2*, and *GFRA1* were determined using MS-HRM.

Supplementary Table S1: The CpG identity and average methylation level of the 36 genes

See Supplementary File S1.

Supplementary Table S2: The Diagnostic performance of selected gene methylation

Gene	AUC	95%CI	Sensitivity (%)	Specificity (%)	Cutoff(β-value)
**GFRA1**	**0.949**	**0.888- 0.982**	**89.06**	**97.56**	**0.160**
**GSTM2**	**0.926**	**0.858- 0.968**	**82.81**	**95.12**	**0.421**
**SEPT9**	**0.877**	**0.798- 0.933**	**71.87**	**97.56**	**0.085**

AUC: area under ROC curve, CI: confidence interval.

Supplementary Table S3: Primer list

name	sequence	product size
MH-GSTM2-F	ATGTTTATGATATTGGGGTATTGGA	228bp
MH-GSTM2-R	TCCTCCTCTCGTTCCTACCTCA	
MH-GFRA1-F	AGTTTAAGGGGGTGTTTGTTGGGT	332bp
MH-GFRA1-R	CATCGATTCCCCCTAACTTAACGACCTC	
GSTM2-F	GAGAAACCAAGTATTTGAGCCCAG	128bp
GSTM2-R	CAGGTCTTGGGAGGAAGCG	
GFRA1-F	GATATATTCCGGGTGGTCCCATTC	150bp
GFRA1-R	GGTGCACGGGGTGATGTACGC	
